# Assessing family medicine trainees – what can we learn from the European neighbours?

**DOI:** 10.3205/zma000963

**Published:** 2015-05-13

**Authors:** Elisabeth Flum, Roar Maagaard, Maciek Godycki-Cwirko, Nigel Scarborough, Nynke Scherpbier, Thomas Ledig, Marco Roos, Jost Steinhäuser

**Affiliations:** 1 University Hospital Heidelberg, Department of General Practice and Health Services Research, Heidelberg, Germany; 2 University of Aarhus, Department of Medical Education, Aarhus, Denmark; 3 Medical University of Lodz, Centre for Family and Community Medicine, Lodz, Poland; 4 Health Education East Midlands, Nottingham, The United Kingdom; 5 Radboud University Medical Centre, Department of Primary and Community Care, Nijmegen, The Netherlands; 6 University of Erlangen-Nuremberg, Institute of General Practice, Erlangen, Germany; 7 University Hospital Schleswig-Holstein, Institute of Family Medicine, Lübeck, Germany

**Keywords:** Primary health car, training, curriculum, competency-based education, assessment

## Abstract

**Background: **Although demands on family physicians (FP) are to a large extent similar in the European Union, uniform assessment standards for family medicine (FM) specialty training and assessment do not exist. Aim of this pilot study was to elicit and compare the different modalities and assessment methods of FM specialty training in five European countries.

**Methods:** A semi structured survey was undertaken based on a convenient sample in five European countries (Denmark, Germany, Poland, the Netherlands and the United Kingdom). The respondents were asked to respond to ten items about aspects of FM specialty training and assessment methods in their respective countries. If available, this data was completed with information from official websites of the countries involved.

**Results: **FM specialty training is performed heterogeneously in the surveyed countries. Training time periods range from three to five years, in some countries requiring a foundation program of up to two years. Most countries perform longitudinal assessment during FM specialty training using a combination of competence-based approach with additional formative and summative assessment. There is some evidence on the assessments methods used, however the assessment method used and costs of assessment differs remarkably between the participating countries.

**Conclusions:** Longitudinal and competence-based assessment is the presently preferred approach for FM specialty training. Countries which use less multifaceted methods for assessment could learn from best practice. Potential changes have significant cost implications.

## Zusammenfassung

**Hintergrund:** In der Europäischen Union sind die Anforderungen seitens der Patienten an die hausärztliche Tätigkeit zu einem hohen Anteil vergleichbar, es gibt jedoch keine einheitlichen Standards für Prüfungen während der Facharzt (FA)-Weiterbildung Allgemeinmedizin (AM) oder bei der Facharztprüfung. Ziel dieser Pilotstudie war es, die verschiedenen Bedingungen und Prüfungsmethoden während der FA-Weiterbildung AM in fünf Europäischen Ländern zu vergleichen.

**Methode:** In fünf Europäischen Ländern (Dänemark, Deutschland, Polen, Niederlande, Groß-Britannien) wurde eine semistrukturierte Befragung in einer Expertenrunde durchgeführt. Die Teilnehmer wurden über verschiedene Aspekte der allgemeinmedizinischen Weiterbildung und Prüfungsmethoden in den jeweiligen Ländern befragt. Falls verfügbar, wurden diese Daten durch Informationen von offiziellen Websites der jeweiligen Länder ergänzt.

**Ergebnisse: **Die Weiterbildung zum FA für AM wird sehr unterschiedlich in den untersuchten Ländern durchgeführt. Die Weiterbildungszeit reicht von drei bis zu fünf Jahren, in manchen Ländern ist vorab ein Fundierungsprogramm von bis zu zwei Jahre verpflichtend. Die meisten Länder erfassen Leistungen longitudinal während der gesamten Weiterbildung, wobei sowohl kompetenzbasierte Ansätze als auch formative und summative Prüfungsmethoden kombiniert werden. Für einige der verwendeten Prüfungsformate ist Evidenz verfügbar. Allerdings unterscheiden sich die Prüfungsmethoden und deren Kosten deutlich zwischen den befragten Ländern.

**Schlussfolgerung: **Longitudinale und kompetenzbasierte Prüfungsansätze werden aktuell in der FA-Weiterbildung AM favorisiert. Länder, die weniger facettenreiche Prüfungsmethoden einsetzen, könnten dabei vom Beispiel anderer Länder lernen. Dabei müssen bei möglichen Veränderungen allerdings die teilweise erheblichen Kostensteigerungen berücksichtigt werden.

## Background

The family physician (FP) is the first medical contact for prevention, acute or chronic diseases and provides longitudinal continuity of comprehensive care in almost all European health care systems [1]. In a changing European Union (EU) with its growing population mobility and doctor migration, continuous health care adaptions are inevitable [2], [3]. Facing these social and economic changes, the European countries have the opportunity to learn from each other [4]. The European Academy of Teachers in General Practice and Family Medicine developed the European Educational Agenda for family medicine (FM) in 2005, defining core characteristics of the discipline and essential competences [5]. This agenda was intended to be incorporated into the national curricula for FM specialty training according to the needs of the particular health care system. Unfortunately, this was not achieved homogenously throughout Europe as several countries are using a different framework model for competency based training [6]. Within the last decades competence-based training has been regarded as an important development within medical education. Compared to traditional education programs, competence-based training aims to be outcome-orientated, analyzing functional occupational roles and assessment of the trainees’ progress. In principle, the competence-based approach could be more flexible, transparent and standardized [7], [8]. The competence-based approach in medical training has already been implemented in European countries such as the Netherlands (NL) and the United Kingdom (UK). Other countries such as Germany (DE) are currently introducing a competency approach for future FM specialty training [6].

So far, there are no studies which examine and compare international ways of qualification and assessment for family medicine.

The aim of this study was to carry out a pilot study aiming to raise different modalities and assessment methods of FM specialty training in five European countries.

## Methods

### Questionnaire

A study group in Heidelberg consisting of three FPs and one FP trainee developed a 10 item semi structured questionnaire addressing aspects of postgraduate training and qualification for FPs. Within these 10 items, prerequisites for admission of examinees to assessment, qualifications of the examiners, evidence for assessment methods and costs of these methods were investigated. The questions were all open-ended and in English. Table 1 (Tab. 1) shows the questionnaire used. Following the professional code of conduct of State Medical Chamber of Baden-Württemberg, for this survey no approval by an institutional research review board was needed [http://www.aerztekammer-bw.de/10aerzte/40merkblaetter/20recht/05kammerrecht/bo.pdf cited 2014 August 12].

Recruitment: The pilot survey was conducted in five European countries based on a convenient sample linked to postgraduate training programs in Denmark (DK), Germany (DE), Poland (PL), the NL and the UK. Target participants for this study were identified due to relationships established via an European implementation research network named “Tailored implementation for chronic diseases” (TICD) [http://www.ticd.umed.lodz.pl/index.php/homepage cited 2014 August 12]. Four researcher of the TICD project were asked to suggest a person in their country who is a well-established experts in the field of FM specialist training. To also include the representation of the European Academy of Teachers in General Practice / Family Medicine (EURACT) a council member of EURACT was additionally asked to participated in the study. Five persons participate in the pilot study, all were FPs and actively involved in FM training and assessment in their respective country. All correspondence was done by email.

#### Data analysis

Data from the questionnaire was analyzed by two researchers from the study group in Heidelberg independently from one another. This extracted data was collated, categorized and then summarized and discussed resulting in a consensus version of results. This version was again double checked by the surveyed participants and the study group. If applicable, information like costs of assessment was expanded from official websites of the participant countries to ensure that the information given was generalizable [http://www.dsam.dk/ cited 2014 August 12], [http://www.sst.dk/ cited 2014 August 12], [http://www.bundesaerztekammer.de/page.asp?his=1.128.129 cited 2014 August 12], [http://www.klrwp.pl/specjalizacja.php cited 2014 August 12], [http://www.leonardo.org.pl/ cited 2014 August 12], [http://www.huisartsopleiding.nl/content.asp?kid=10000962&fid=-1&bid=10118723 cited 2014 August 12], [http://www.rcgp.org.uk/ cited 2014 August 12]. Special emphasis was set on assessment methods and organization of FM specialty training. The enhanced version of results was then revised by each member of the study group in Heidelberg to ensure correct reproduction of data, resulting in a final version, which provided a descriptive overview of FM specialty training and assessment methods in the different countries. All indicated amounts of money were converted into US dollars (US$) according to currency exchange rates on 30.01.2014.

## Results

All five experts in the field of FM specialist training agreed to participate. The duration of FM specialty training in the participating countries ranges from three to five years. All FM specialty trainings include hospital and practice training in approved training posts. In DK and the UK, a foundation program - a compulsory medical training part in between graduation and start of FM specialty training - is mandatory.

As the FM training is highly favored in the UK and in the NL, a national selection process before training was implemented in the UK and soon will be in the NL.

The contents of FM specialty training are defined by FP specialist representatives at national level in all surveyed counties but DE, where all medical specialties not only FPs determine the details.

In all participant countries, the assessment is carried out and results are accepted nationwide. All countries had a longitudinal assessment continuously during the whole duration of FM specialty training except DE, which has one final 30 minute-theory oral exam at the end of scheme. Topics examined are not particularly specified or standardized and depend therefore on the examiners. DK [http://www.dsam.dk/flx/english/hippokrates/denmark/ cited 2014 November 20] and the NL perform a competency-based longitudinal assessment of trainees combined with formal exams as Multiple Choice Questions (MCQ) and assessment of communication skills in the NL. The UK has an elaborate assessment method, it consists of three parts: Applied Knowledge Tests (AKT), Clinical Skills Assessments (CSA) and Workplace Based Assessments (WPBA, assessment of performance in everyday clinical practice to integrate clinical knowledge and skills), which is continuously carried out through specialty training. These three parts combine multiple assessment methods as computer based MCQ exams and the use of electronic devices in testing, as well as assessment of competencies and procedural skills in different settings as live observation and video consultation with “real” or simulated patients. Special emphasize is set on feedback from colleagues and the patients’ perspective. Trainees have to keep a detailed e-portfolio, which includes a learning log, a personal development plan, regular self-ratings and educator reviews. 

In the NL and the UK there is robust evidence for the reliability and validity of the different assessment methods, whereas there is less evidence for the assessment methods used in DE, DK and PL [9], [10], [11], [12], [13], [14], [15], [http://www.rcgp.org.uk cited 2014 August 12]. The process of qualification of examiners differs also from only formal qualifications of examiners in DE, to facultative trainers’ courses in PL and mandatory trainers’ courses in DK, the NL and the UK.

While in DK and the NL no detailed information about costs of assessments are available, trainees in DE are charged up to US$ 341 for the final exam in total. In the UK, trainees have to pay separate fees for mandatory registration with the RCGP (US$ 2.036), AKT (US$ 835) and CSA (US$ 2.807). Reimbursement of examiners varies from US$ 102 in PL and US$ 205 in DE up to US$ 651 in the UK.

For details see table 2 (Tab. 2) and table 3 (Tab. 3).

## Discussion

This pilot study gives an overview of specialty training and different qualification assessment methods for FM in five European countries. Assessment methods differ remarkably in all surveyed countries. All countries but DE perform longitudinal assessment during FM specialty training using a combination of a competence-based approach with additional formative (formal and informal assessment methods during the whole period of training learning, “assessment for learning”) and summative assessment (formal assessment of outcome, summarization learning progress at a particular time, “assessment of learning”). DK and the NL assess all required competencies longitudinally in an ongoing process during the whole training, including in the NL communication skills. The process of qualification for FM in the UK is highly structured. In contrast, DE uses no longitudinal assessment and only one final theoretical oral assessment at the end of specialty training.

Evidence published to date validates the knowledge test, the communication skills test and the competency assessment list, “Compass” in the NL [9], [10], [11], [12], [13] and assessment methods in the UK [14], [15], [[http://www.rcgp.org.uk cited 2014 August 12]. Otherwise, many FM assessment methods have been reported in scattered surveys, although final conclusions or recommendations are yet to emerge [16].

As outlined previously, population mobility and physician migration in the EU presents new challenges for health care. For quality and patient safety reasons, competence profiles and future assessment methods in the EU might preferably be standardized [2], [17]. If this were to be the case, competencies requirements could still differ between countries and individuals, but an exchange of physicians and trainees between countries would be more effectively facilitated. In particular DE, performing the least multifaceted assessment of all surveyed countries, could follow the different European examples and implement a longitudinal assessment of competences and skills in FM specialty training. In DE there is evidence, that nearly every fourth of the FM-trainers do give informal feedback less than once per month to their trainees [18]. It should be taken into consideration that critics claim that the competence-based approach neglects expertise and raises negative pressure on examiners and examinees [16]. So far, little evidence exists that performance in the different assessment methods (MCQ, video or live observation, OSCE, etc.) correlates with clinical skills in real working conditions [19], [20], [21]. Therefore, countries with rather elaborate specialty training may continuously reflect on their methods of assessment.

There is evidence that assessment influences learning [22], [23], therefore assessment may be an instrument for the regulation of content of FM specialty training and should match population health needs as well as learning objectives and curriculum. To assess the individual level of professional competence, a multifaceted and complex assessment has to be performed [24]. Preferably, the assessment should be an integrated part of the curriculum using a blend of methods [25].

As shown in the results, structured and standardized assessment methods are considerably more costly than less multifaceted approaches, differing up to more than US$ 3.000 per examinee. Therefore, in each European country, available personal and financial resources have to be considered, too. It remains a subject for discussion how much FM specialty training may cost and to what extend examinees may be charged. However, FM trainers should receive professional recognition including adequate reimbursement.

The strength of the study is that it is a first overview of different methods of specialty training and qualification assessment for FM in Europe. The pilot survey provides baseline data on an area that is likely to become more topical in the context of EU policy. A limitation of the study is the convenience sampling. However, it was in line with the aim of the study to gather and compare baseline information on the variability and diversity of FM specialty training and assessment methods within the participating countries. The questionnaire contained furthermore some closed-ended questions, which may have led to limited answers. In addition, the data collected in the sample was heterogeneous. This was somewhat challenging for summarizing in the results section. Moreover, through the “coding” process of summarizing answers to the open-ended questionnaire, some subjective bias may occur. To minimize this effect, all authors thoroughly reviewed the transcript manuscript. Finally, evidence-based information is not available for all questions in the questionnaire, which may lead to expert-based conclusions being drawn.

Next work package will be to build a questionnaire with the results of this pilot-study to raise quantitative data about FM specialty assessment throughout Europe. 

## Conclusion

This pilot study provides a first overview of different methods of FM specialty training and assessment methods in Europe. Longitudinal and competence-based approaches are presently favored in developed European countries. Countries as DE, which perform less multifaceted assessments can benefit from best practice in neighboring countries. Of critical importance to future FM specialty training programs is the financial aspect: assessing competencies is more costly than assessing knowledge. More research is needed, to establish whether performance in assessment correlates with clinical skills in real working conditions and the new challenges occurring in primary health care service delivery. 

## Authors' contributions

EF and JS had been involved in conception and design and analysis of data. RM, MGC, NiS, NS, TL, MR made substantial contributions to acquisition and interpretation of data. All authors have been involved in revising the manuscript thoroughly and have given final approval of the version to be published.

## Acknowledgements

This project was supported by the Ministry of Labour and Social Affairs, Families, Woman and Senior Citizens by means from the federal state of Baden-Württemberg. We would like to thank our colleague and native English speaker Sarah Berger for editing the manuscript.

## Competing interests

The authors declare that they have no competing interests.

## Figures and Tables

**Table 1:**
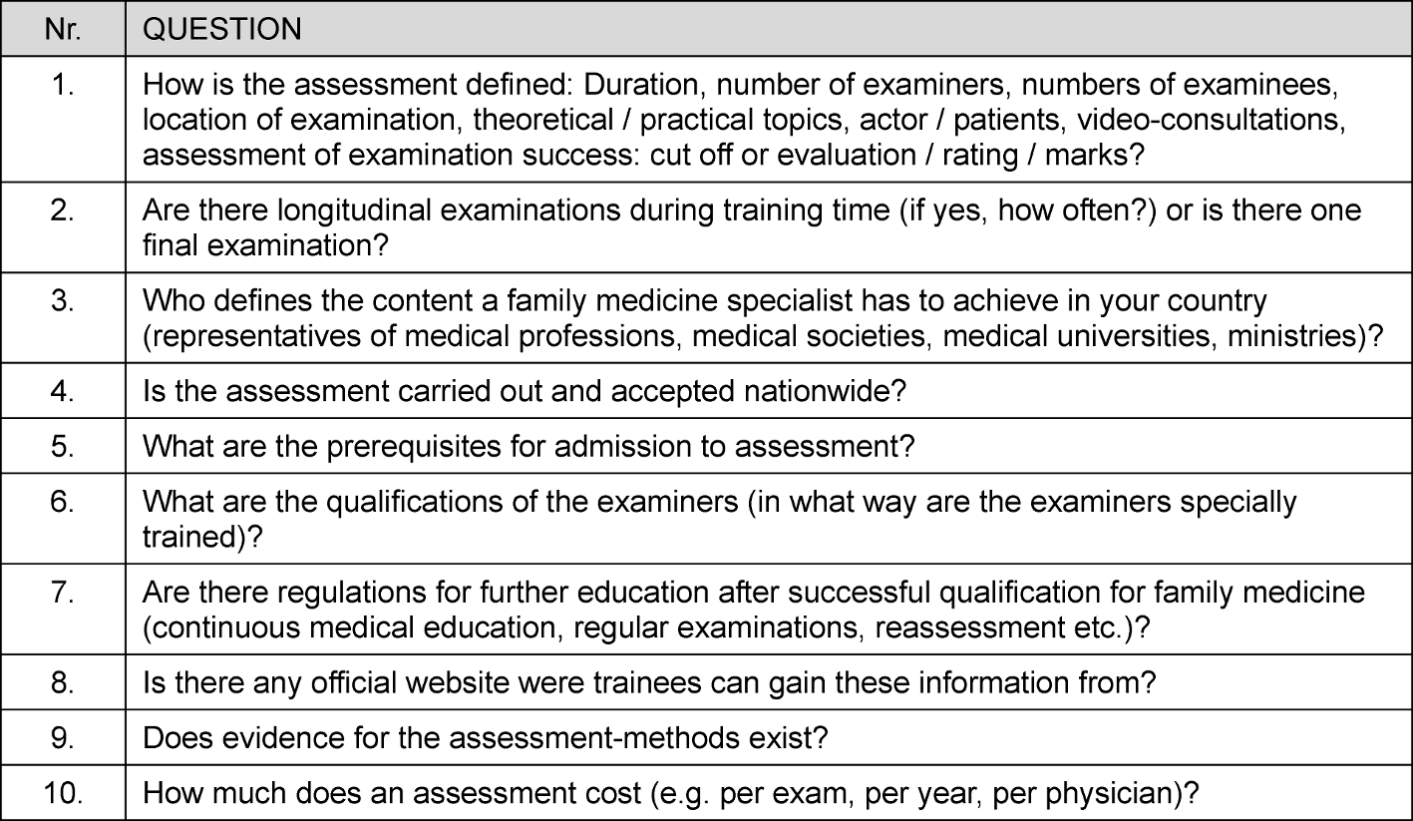
Questionnaire

**Table 2:**
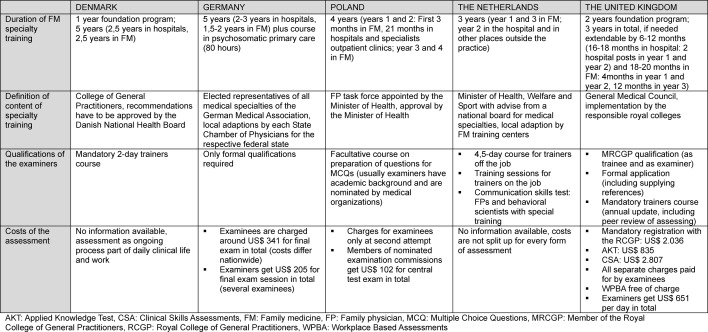
General aspects of FM specialty training in international comparison

**Table 3:**
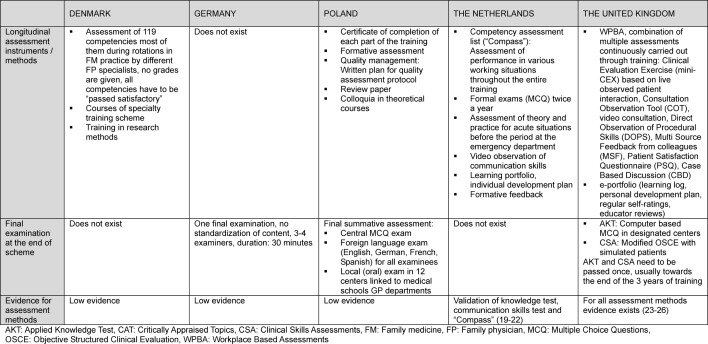
Assessment during FM specialty training in international comparison
